# Cross-sectional analysis of the reporting of continuous outcome measures and clinical significance of results in randomized trials of non-pharmacological interventions

**DOI:** 10.1186/1745-6215-15-362

**Published:** 2014-09-17

**Authors:** Tammy C Hoffmann, Sarah T Thomas, Paul Ng Hung Shin, Paul P Glasziou

**Affiliations:** Centre for Research in Evidence-Based Practice, Faculty of Health Sciences and Medicine, Bond University, University Drive, Robina, Gold Coast, Australia; School of Health and Rehabilitation Sciences, The University of Queensland, Brisbane, Australia

**Keywords:** RCT, Continuous outcome measure, Clinical significance, Scoring, Reporting, CONSORT

## Abstract

**Background:**

Reporting the scoring details of continuous outcome measures in randomized trials allows readers to interpret the size of any effect of the intervention. This study aimed to determine, in a sample of randomized trials: 1) the completeness of reporting of scoring details for continuous outcome measures, and 2) whether trial authors comment on the clinical significance of statistically significant trial results.

**Methods:**

A descriptive analysis of randomized trials of non-pharmacological interventions published during 2009 in the six leading general medical journals (n = 138), and which used at least one continuous outcome measure (n = 85). From each trial report, two authors independently extracted the following information about each continuous outcome measure: the reporting of its scoring details, presentation of its results, and the reporting and justification of the clinical significance of the results.

**Results:**

Across the 84 trials, we identified 336 continuous outcome measures. A total of 146 (44%) were published measures, 12 (4%) were adapted from published measures, 5 (1%) were developed for the trial, and 173 (51%) were ‘conventional measures’ for which scoring details are not necessary (such as weight). For 57 (35%) of the 163 non-conventional outcome measures no scoring details or reference to the outcome measure were provided in the trial report. Of the 159 outcome measures with a statistically significant result, clinical significance was not mentioned for 81 (51%) and was reported without any elaboration or justification for 39 (25%) of them.

**Conclusions:**

Scoring details of continuous outcome measures used in this sample of randomized trials of non-pharmacological interventions were incompletely reported, which hampers interpretation of a trial’s results. Complete reporting of scoring details is important when considering the clinical significance of the results. When deciding about an intervention, having this information may help clinicians in their conversations with patients about the possible benefits and harms, and their size, of the intervention.

**Electronic supplementary material:**

The online version of this article (doi:10.1186/1745-6215-15-362) contains supplementary material, which is available to authorized users.

## Background

Randomized trials are widely used to evaluate the effectiveness of an intervention. All randomized trials assess endpoint variables using outcome measures, which enables comparison of groups in the trial. These outcome measures need to be carefully selected and reported as they can influence the conclusions of the trial and the usability of its results.

Outcomes can be either dichotomous (such as dead/alive and readmitted/not readmitted) or continuous (such as weight and level of pain). Some continuous outcomes (such as height, length of stay, and blood pressure) are likely to be familiar to most readers of trial reports and their scoring requires minimal description. However, many continuous outcomes are assessed using measures for which interpretation of the results relies on having at least some understanding of how the measure is scored, such as the possible range of scores. Without this information, understanding the meaning of an effect size is hampered. For example, the clinical significance of a two-point reduction on a 10-point scale is very different to the clinical significance of a two-point reduction on a 100-point scale.

Readers of trials can sometimes mistakenly assume that clinical trial results which are statistically significant are also clinically significant [1]. Providing sufficient details about the scoring of continuous outcome measures allows readers to interpret the effect size of the intervention and the clinical significance of the result, as opposed to relying solely on the interpretation of the authors. Adequate reporting of results, which includes information such as confidence intervals of effect sizes, can also help with deliberations about the clinical meaningfulness of trial results.

Previous studies of the quality of continuous outcome measure reporting have primarily focussed on publication bias [2, 3] and the suitability and reporting of the psychometric properties of the measures [4, 5]. The reporting of scoring and interpretation details for continuous outcome measures in randomized trials has not been investigated. This study aimed to determine, in a sample of randomized trials of non-pharmacological interventions: 1) the completeness of reporting of scoring details for continuous outcome measures, and 2) whether trial authors comment on the clinical significance of statistically significant trial results.

## Methods

### Design

We performed a descriptive cross-sectional analysis of randomized controlled trials which used at least one continuous outcome measure.

### Search strategy and selection of reports of trials

Trials were screened for eligibility from a previously collected sample [6] of reports of randomized controlled trials of non-pharmacological interventions published in 2009 in one of the six leading general medical journals (based on ISI Web of Knowledge Impact Factor for 2010) - *New England Journal of Medicine,* the *Journal of the American Medical Association, Lancet, Annals of Internal Medicine, PLOS Medicine,* and the *British Medical Journal*. An experienced medical librarian searched PubMed in April 2011, using the restrictions of year (2009), publication type (‘randomized controlled trial’), and journal title (the six chosen journals). We supplemented our electronic search by hand-searching the table of contents for these journals for 2009. Two authors (TH and PG) screened the 358 titles and abstracts retrieved, identified reports that might meet the inclusion criteria, and retrieved the full-text (n = 138). From this sample, the full-text of each trial was examined by two authors (BNHS and ST) and all trials which had used at least one continuous outcome measure were included (n = 84) in the current study (for categories of interventions evaluated by these trials, refer to Additional file 1: Table S1).

### Data extraction and analysis

For each included trial, two authors (BNHS and ST) independently extracted information about each continuous outcome measure using a data extraction form developed in Microsoft Excel. Data were extracted as to whether the outcome measure was: a specified primary outcome measure; a published measure and if so, was a citation provided and/or were scoring details provided in the paper; a measure adapted from a published measure and if so, were details of the adapted scoring provided; a measure developed for the study and if so, were scoring details provided; or a measure for which scoring details are not necessary (such as length of stay or weight) and referred to in this paper as a ‘conventional’ measure. Throughout this paper, by scoring details we mean details which assist in interpretation of the score (for example, the total possible score, subscale scores where relevant, score range, and/or explanation of the anchor points such as whether a high score means better or worse). We do not mean details about how to administer or score the outcome measure or its psychometric properties. For each measure for which scoring details were provided in the paper, the location of this information in the paper was recorded. Data about the summary measures (such as mean or effect size) and precision estimates (such as confidence intervals) used to report results were extracted for all included outcome measures. Any words about clinical significance (or related terms, such as ‘clinically worthwhile’ or ‘minimum clinically important difference’) were searched for in the PDF of each trial and recorded. It was also noted which outcome this was referring to, if a statistically significant effect (as defined by the trial authors) had been reported for that outcome, and if any justification or explanation for referring to the effect as clinically significant was provided. Discrepancies between raters were resolved by discussion and a consensus rater (TH) was used when agreement could not be met. Data were analysed descriptively.

## Results

### Categories of outcome measures and their scoring details

**Figure 1:**
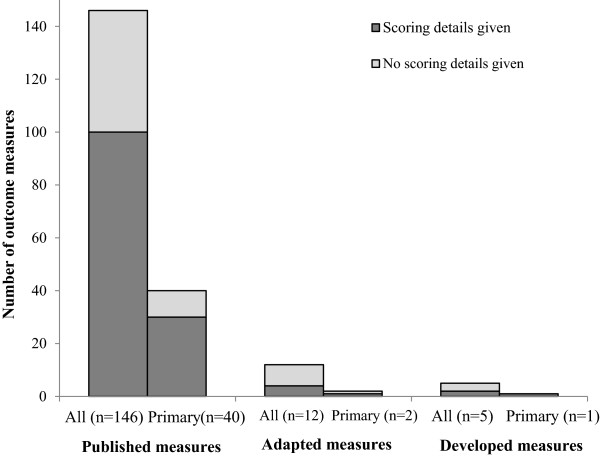
**Number and percentage of published, adapted, and developed outcome measures for which scoring details were provided (for all continuous outcome measures and for only primary outcome measures).**

Across the 84 trials, a total of 336 continuous outcome measures were used, with a mean of 4 continuous outcome measures per trial (SD 2.6, range 1 to 11). Of these, 88 (26%) were specified as primary outcome measures, 245 (73%) were specified as secondary outcome measures, and 3 (1%) were not classified as either by trial authors.Half of all the outcome measures (173, 51%) and primary outcome measures (45, 51%) were conventional measurements for which reference or scoring details are not required. Of the remaining 163 outcome measures, either no scoring details or reference to the outcome measure were provided for 57 (35%) of the measures (Figure 1). For the 43 primary outcome measures, either no scoring details or reference to the outcome measure were provided for 12 (28%) of the remaining (Figure 1). Of the 146 outcome measures that were reported to be a published outcome measure, at least a reference to the measure was provided for 140 (96%). Similarly, of the 40 primary outcome measures which were a published measure, a reference to the measure was provided for 39 (98%) of them.

Below are some verbatim examples from papers where outcome measures were listed and a reference provided, but no scoring details or score interpretation were provided:

- In Methods: ‘Our secondary outcomes included the state trait anxiety inventory [reference provided]…’. In Results: ‘…61 (21%) women in the intervention group had a score >44 on the state-trait anxiety inventory compared with 86 (27%) in the control group…’
- In Methods: ‘We used the paediatric quality of life inventory 4.0 (PedsQL(4.0) [reference provided] to measure state of wellbeing or quality of life.’
- In Methods: ‘Secondary outcomes were … health status (the short-form [SF-12] mental subscore) [reference provided] and quality of life (the EuroQol [EQ-5D]) [reference provided] analysed as continuous variables at 4 and 8 months.’ In Results: ‘…adjustment for baseline imbalance increased the comparisons (e.g., for the SF-12 outcome the difference was then 4.0, 95% CI 0.1-8.0; p = 0.045).’
- In Methods: ‘The primary outcomes were the scores on the Barthel index and the Rivermead mobility index [reference provided]’ ‘A 2 point change on the Barthel index was thought to be a meaningful change in independence with respect to activities of daily living.’ In Abstract: ‘…no significant differences were found in mean Barthel index scores at six months post-randomisation between treatment arms (mean effect 0.08, 95% confidence interval -1.14 to 1.30; P = 0.90)… Similarly, no significant differences were found in the mean Rivermead mobility index scores between treatment arms (0.62, -0.51 to 1.76; P = 0.28)…’

### Location of scoring details in the paper

Table 1 shows where scoring details were located in the paper when they were reported. For all measures and primary outcome measures only, this information was most often found in the methods section (37% and 54% respectively) of the paper.

**Table 1:** **Location of scoring details in the paper for measures which reported scoring details**

Location in paper	All outcome measures n = 106	Primary outcome measures n = 31
Methods section	45 (42)	20 (65)
Results - in table, figure, or footnote to these	35 (33)	2 (6)
In more than one place in paper	33 (31)	12 (39)
Abstract	3 (3)	2 (6)
Discussion section	2 (2)	1 (3)

Results are presented as numbers (percentages).

Totals are >100% as location was more than one place in some papers.

### Reporting of results

For 238 (71%) of the outcome measures, a mean and a measure of variance (such as standard deviation) was reported. For 11 (3%) of the measures, only an effect size was reported. Confidence intervals were reported as part of the results for 237 (71%) of all outcome measures and for 73 (83%) of the 88 primary outcome measures.

### Reporting of the clinical significance of the results

A statistically significant result was reported for 159 (47%) of the 336 continuous outcome measures and 61 (75%) of the 88 primary outcome measures. For these measures, Table 2 shows whether there was any comment about the clinical significance of these results in the trial report and if so, any elaboration about how clinical significance was determined. There was no comment on clinical significance for 51% of all outcome measures and 30% of primary outcome measures. Following are two verbatim examples which discuss clinical significance with some justification of these:

**Table 2:** **Reporting and elaboration of clinical significance of results which were reported as statistically significant**

Reporting of clinical significance of statistically significant results	All outcome measures reporting a statistically significant result n = 159	Primary outcomes reporting a statistically significant result n = 61
Clinical significance commented on and reference or justification provided	39 (25)	26 (43)
Clinical significance commented on, no justification provided	39 (25)	17 (28)
No comment on clinical significance of results	81 (51)	18 (30)

Results are presented as numbers (percentages).

- *Example 1* [7]: ‘The minimal clinically important difference for Roland Morris Disability Questionnaire (RMDQ) score ranges from 2 to 8 and is dependent on baseline score.^17,51,52^ The minimal clinically important difference for RMDQ score may be as low as 2 for subpopulations with high rates of chronicity,^52^ as would be the case with our patient population.’
- *Example 2* [8]: ‘Minimally clinically important differences…have been estimated for several of the outcome measures used in this study.[…] estimated minimally clinically important differences have ranged between 3.5 and 4.3 points for the SF-36 PCS,^25^ 1.0 and 2.5 points for back pain,^15,25^ and between 2 and 3 points for the Roland–Morris scale.^16^ The SF-36 physical function and EQ-5D estimates are 15 and 0.08 points, respectively.^26,27^’

For 177 outcome measures, the authors reported that the results were not statistically significant, and for 10 of these (eight trials), the trials contained a comment about the clinical significance or meaningfulness of these results. For seven measures, the comment appropriately referred to between-group differences (including confidence intervals of the difference) and the absence of a clinically meaningful difference between the trial’s groups. For three measures, comments referred to within-group changes as being clinically meaningful (despite between-group differences that were not statistically significant).

## Discussion

In this study, we identified 336 continuous outcome measures across 85 randomized trials of non-drug interventions and found incomplete reporting of scoring details of outcome measures and clinical significance. Scoring details were not given for 35% of all non-conventional outcome measures. Clinical significance was not mentioned, or reported without justification, for 76% of all the outcome measures that had a statistically significant result.

The strengths of this study include the wide range of non-drug interventions and outcome measures in the sample of included papers and the independent data extraction by two assessors. A limitation is that the sample of trials only included non-pharmacological interventions and our findings may not extend to trials of pharmacological interventions. Our sample of trials was drawn from the top six general medical journals and as the quality of reporting is generally better in these journals [9], our study is therefore likely to have underestimated the size of the problem. Our sample of trials was from 2009 and so reporting quality may have changed since then, although there have been no explicit strategies since then that have been aimed at improving the reporting of continuous outcome measures that we are aware of. Additional research into the reporting of continuous outcome measures and their scoring in a broader sample of trials would enable further understanding of the extent of this problem. Another limitation is that we did not check whether the published references provided for outcome measures did indeed contain scoring details. Finally, we only recorded whether authors had provided a justification for their interpretation of clinical significance, we did not assess whether the justification was appropriate. Likewise, we did not assess the risk of bias in each trial and whether authors’ claim of statistical significance were appropriate.

To the best of our knowledge, this is the first study specifically looking at the reporting of scoring details of continuous outcome measures in randomized trials. Reporting of clinical significance has been analysed in a small number of studies [10–13], with under-reporting found. Chan *et al*. [10] analyzed the reporting of clinical significance in a randomly selected sample of 27 randomized trials, which consisted of a mixture of pharmacological and non-pharmacological interventions. The categories of outcome measures (such as continuous or other) that were used in their included trials are not described. They found that clinical significance was mentioned in 20 (74%) trial reports, with trial authors not providing a justification for their clinical interpretation in most (15, 75%) of these [10]. Chan *et al*. also assessed the quality of reporting of factors related to clinical significance (such as clearly defined primary outcome and reporting of confidence intervals) and concluded that authors often fail to report sufficient information that would allow readers to interpret the study results from their own perspective. For example, confidence intervals were reported for only 41% of primary outcomes. In a systematic review of pharmacological interventions for dementia, it was found that only 46% of the 57 included randomized trials discussed the clinical significance of the results [12].

Incomplete reporting restricts the usability of published research and contributes to the problem of waste in research [14]. If scoring details are not provided for published measures, it is, at best, inconvenient as the research user needs to look up the scoring details in the cited reference or independently locate the reference for the measure. While this does not render the research unusable, it does slow down the process of using evidence and introduces an unnecessary obstacle for the evidence user. However, if no scoring details are provided for adapted or newly developed measures, the only way to obtain the missing information is by contacting trial authors. This is a time-consuming step which few clinicians have time to undertake and for which a response is not guaranteed. Without this complete information, the reader is unable to fully interpret the results of the study.

The CONSORT (Consolidated Standards of Reporting Trials) statement was developed to guide authors on reporting the details of a randomized trial [15]. When the trials included in this study were published, the available CONSORT statement was the 2001 version. It states in Item 6A (clearly defined primary and secondary outcomes): ‘Where available and appropriate, previously developed and validated scales or consensus guidelines should be used… Authors should indicate the provenance and properties of scales’ [16]. In the elaboration of this item, it advocates for the use of published or existing measures. However, it does not explicitly advise authors of trials to provide scoring details of the measure in the paper. The lack of specific instruction to do so may be contributing to the incomplete reporting of such details by authors. In the revised 2010 CONSORT statement [15], there has been some minor rewording of Item 6A and it now states that: ‘Completely defined pre-specified primary and secondary outcome measures’ should be reported, ‘including how and when they were assessed’. However, it is not clear whether ’completely defined’ also encompasses scoring details and/or ranges for continuous outcome measures.

The clinical meaning of intervention effects cannot be ascertained by the researcher alone, and, as some argue, nor by the clinician alone. Extrapolating the clinical meaning of a result requires careful consideration of the benefits of the intervention versus the possible burden on the patient, and this balance varies from one patient to another [17]. Various methods of determining clinical significance have been proposed [18, 19] but there is no consensus on the ideal method of doing this. For many interventions, the minimal clinically important difference (MCID) that is considered acceptable varies between patients [20] and between patients and clinicians [21]. Various terms (such as clinical significance, clinical meaningfulness, clinically worthwhile, and MCID) are also used interchangeably which can further complicate interpretations of results that are made by authors. In many situations, it may be that a patient-centred approach to determining the clinical meaningfulness of a result is best. In this approach, the possible benefits of the intervention, as well as their size or likelihood are communicated to patients, along with any possible harms (and their size or likelihood), costs, and inconveniences of the intervention. Only then can patients have an informed discussion with their clinician about whether the likely benefit from the intervention outweighs the likely harm to an extent that is acceptable to them. Importantly, this shared decision-making approach also enables incorporation of the patient’s preferences and values into the discussion, with each patient approaching and managing the benefit-harm trade-off of a decision differently. Complete reporting of scoring details of continuous outcome measures in trial reports enables clinicians to incorporate information about the likely size of an intervention’s benefit and/or harm when discussing a particular intervention with patients.

## Conclusions

The scoring details of some continuous outcome measures used in this sample of randomized trials of non-pharmacological interventions in general medical journals were incompletely reported. Authors of trials should ensure that measures and their scoring details are described in sufficient detail in the trial report to facilitate interpretation of the results. This additionally enables clinicians to have this information at hand when considering the clinical significance of the results. When deciding about an intervention, this information may assist clinicians in their conversations with patients about the possible benefits and harms, and their size, of the intervention.

## Electronic supplementary material

Additional file 1: Table S1: Categories of interventions evaluated in sample of trials (n = 84). (DOCX 14 KB)

## Abbreviations

CONSORT: Consolidated Standards of Reporting Trials
MCID: Minimum clinically important difference.

## References

[1] Freiman JA, Chalmers TC, Smith H, Kuebler RR (1978). The importance of beta, the type II error and sample size in the design and interpretation of the randomized control trial. Survey of 71 “negative” trials. New Eng J Med.

[2] Savage DF (2010). Reporting of continuous outcome measures in randomized clinical trials: is the whole story being told?. J Invest Med.

[3] Schriger DL, Savage DF, Altman DG (2012). Presentation of continuous outcomes in randomised trials: an observational study. BMJ.

[4] Baker GA, Hesdon B, Marson AG (2000). Quality-of-life and behavioral outcome measures in randomized controlled trials of antiepileptic drugs: a systematic review of methodology and reporting standards. Epilepsia.

[5] Geyh S, Cieza A, Kollerits B, Grimby G, Stucki G (2007). Content comparison of health-related quality of life measures used in stroke based on the international classification of functioning, disability and health (ICF): a systematic review. Qual Life Res.

[6] Hoffmann TC, Erueti C, Glasziou PP (2013). Poor description of non-pharmacological interventions: analysis of consecutive sample of randomised trials. BMJ.

[7] Dobscha SK, Corson K, Perrin NA, Hanson GC, Leibowitz RQ, Doak MN, Dickinson KC, Sullivan MD, Gerrity MS (2009). Collaborative care for chronic pain in primary care: a cluster randomized trial. JAMA.

[8] Wardlaw D, Cummings SR, Van Meirhaeghe J, Bastian L, Tillman JB, Ranstam J, Eastell R, Shabe P, Talmadge K, Boonen S (2009). Efficacy and safety of balloon kyphoplasty compared with non-surgical care for vertebral compression fracture (FREE): a randomised controlled trial. Lancet.

[9] Peron J, Pond GR, Gan HK, Chen EX, Almufti R, Maillet D, You B (2012). Quality of reporting of modern randomized controlled trials in medical oncology: a systematic review. J Natl Cancer Inst.

[10] Chan KB, Man-Son-Hing M, Molnar FJ, Laupacis A (2001). How well is the clinical importance of study results reported? An assessment of randomised controlled trials. CMAJ.

[11] Moher D, Dulberg CS, Wells GA (1994). Statistical power, sample size, and their reporting in randomized controlled trials. JAMA.

[12] Molnar FJ, Man-Son-Hing M, Fergusson D (2009). Systematic review of measures of clinical significance employed in randomized controlled trials of drugs for dementia. J Am Geriatr Soc.

[13] Pocock SJ, Geller NL, Tsiatis AA (1987). The analysis of multiple endpoints in clinical trials. Biometrics.

[14] Chalmers I, Glasziou P (2009). Avoidable waste in the production and reporting of research evidence. Lancet.

[15] Schulz KF, Altman DG, Moher D, Group C (2010). CONSORT 2010 statement: updated guidelines for reporting parallel group randomised trials. BMJ.

[16] Altman DG, Moher D, Schulz KF (2001). The CONSORT statement: revised recommendations for improving the quality of reports of parallel-group randomised trials. Lancet.

[17] Rothwell PM (2006). Factors that can affect the external validity of randomised controlled trials. PLoS Clin Trials.

[18] Beaton DE, Boers M, Wells GA (2002). Many faces of the minimal clinically important difference (MCID): a literature review and directions for future research. Curr Opin Rheumatol.

[19] Man-Son-Hing M, Laupacis A, O’Rourke K, Molnar FJ, Mahon J, Chan KY, Wells G (2002). Determination of the clinical importance of study results. J Gen Intern Med.

[20] Beaton DE, Tarasuk V, Katz JN, Wright JG, Bombardier C (2001). Are you better?” A qualitative study of the meaning of recovery. Arthritis Rheum.

[21] McAlister RA, O’Connor A, Wells GA (2001). When should hypertension be treated? The different perspectives of Canadian family physicians and patients. CAMJ.

