# Supplementary material for: Cross-sectional analysis of the reporting of continuous outcome measures and clinical significance of results in randomized trials of non-pharmacological interventions
Source: Trials. 2014 Sep 17;15:362. doi: 10.1186/1745-6215-15-362 (PMC4177425; doi:10.1186/1745-6215-15-362)
Supplement: Supplementary file 1 — Additional file 1: Table S1: Categories of interventions evaluated in sample of trials (n = 84). (DOCX 14 KB) [file 13063_2014_2232_MOESM1_ESM.docx]

**Additional file 1: Table S1 Categories of interventions evaluated in sample of trials (n = 84)**

| **Categories of interventions** | n (%) |  |
| --- | --- | --- |
| Education and training | 14 (17) |  |
| Device | 13 (15) |  |
| Complex intervention | 13 (15) |  |
| Diet | 11 (13) |  |
| Exercise or physical therapy | 10 (12) |  |
| Surgery or perioperative intervention | 10 (12) |  |
| Service delivery | 4 (5) |  |
| Other | 4 (5) |  |
| Psychosocial intervention | 4 (5) |  |
| Complementary and alternative therapy | 1 (0.01) |  |
